# Supplementary material for: A randomized controlled trial of the effectiveness of a community-based rabies vaccination strategy
Source: bioRxiv. 2024 Oct 31:2024.10.28.620430. Preprint. [Version 1] doi: 10.1101/2024.10.28.620430 (PMC11565783; doi:10.1101/2024.10.28.620430)
Supplement: Supplement 1 [file media-1.pdf]

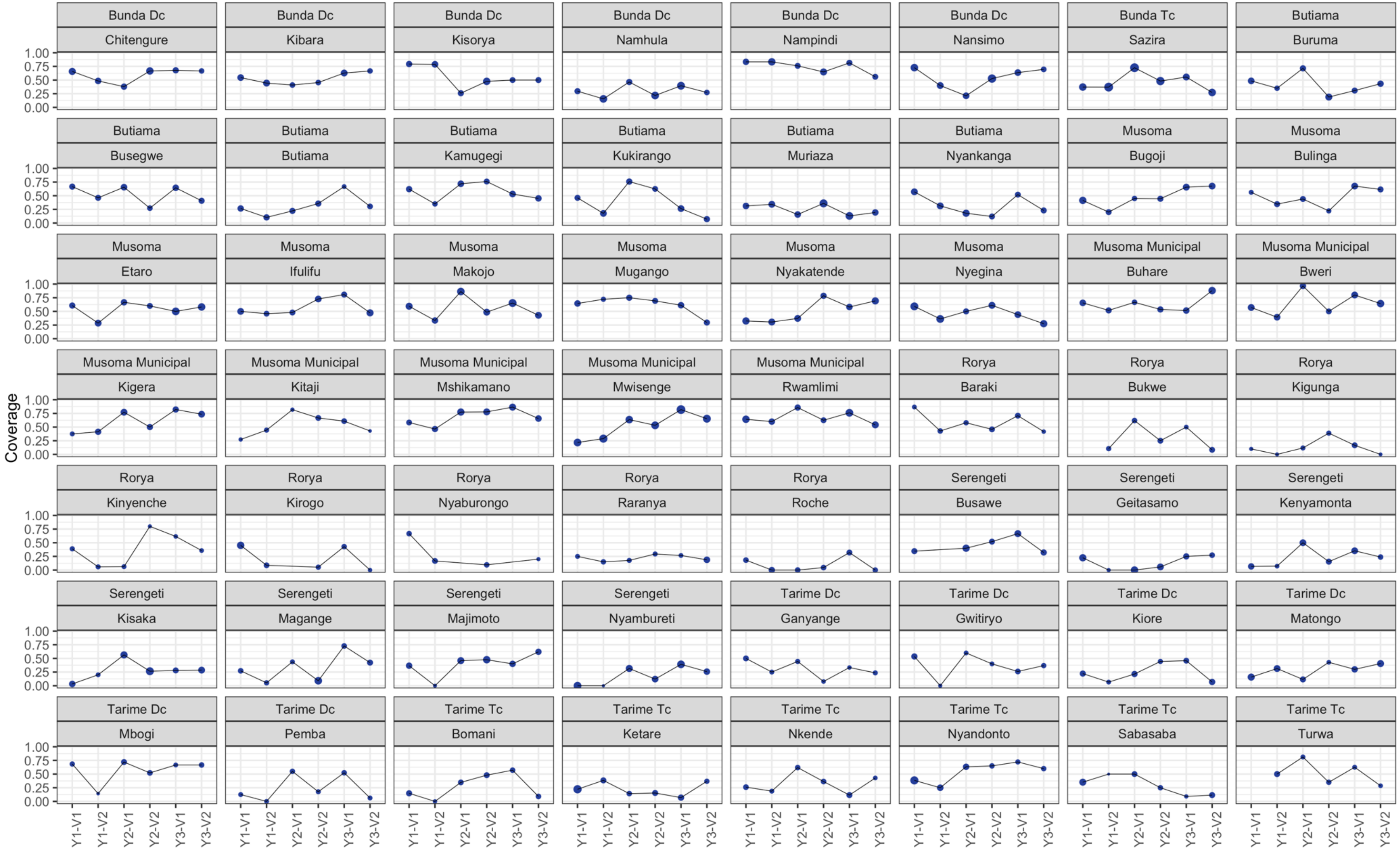

Figure S1. Coverage by ward and survey time point in the Team-based arm. Point area is proportional to the number of dogs surveyed.
