# Supplementary material for: A randomized controlled trial of the effectiveness of a community-based rabies vaccination strategy
Source: bioRxiv. 2024 Oct 31:2024.10.28.620430. Preprint. [Version 1] doi: 10.1101/2024.10.28.620430 (PMC11565783; doi:10.1101/2024.10.28.620430)
Supplement: Supplement 3 [file media-3.pdf]

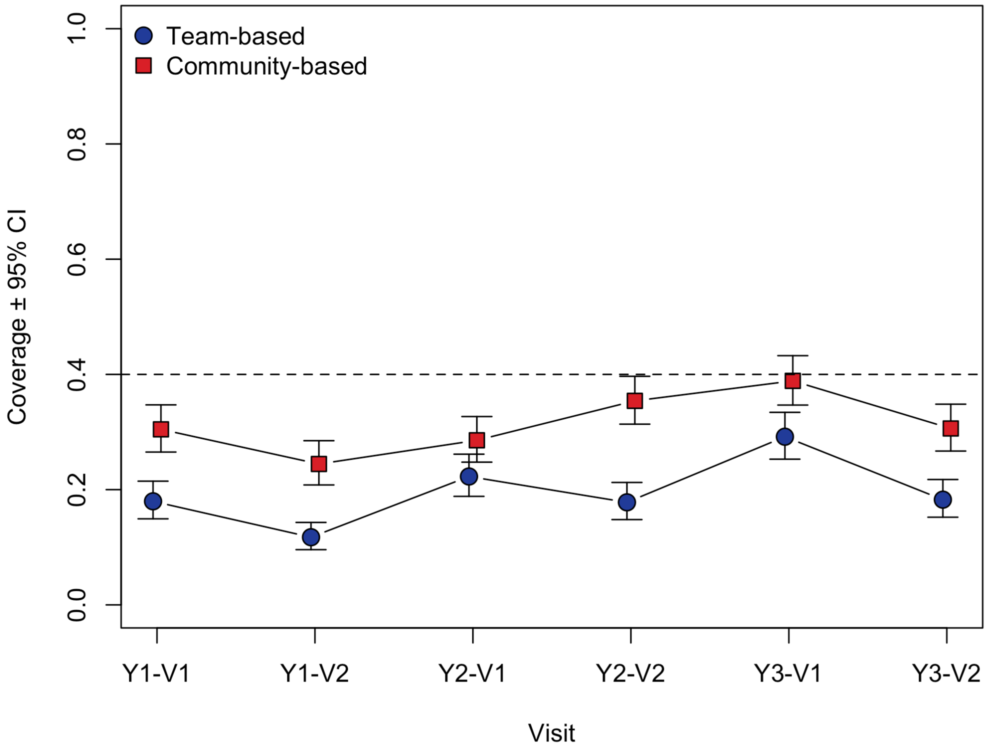

Figure S3. Estimated coverage ± 95% confidence limits at each survey time point, by trial arm. A more stringent definition of coverage was used, where dogs claimed to be vaccinated but where no vaccination certificate could be produced were assumed to be unvaccinated.
