# Supplementary material for: A randomized controlled trial of the effectiveness of a community-based rabies vaccination strategy
Source: bioRxiv. 2024 Oct 31:2024.10.28.620430. Preprint. [Version 1] doi: 10.1101/2024.10.28.620430 (PMC11565783; doi:10.1101/2024.10.28.620430)
Supplement: Supplement 5 [file media-5.pdf]

Table S1. Summary of methods used for primary and secondary analyses. Generalized linear mixed-effects models (GLMMs) were fitted by maximum likelihood (ML) using the glmmTMB package and by Monte Carlo Markov-Chain (MCMC) using the brms package.

Analysis	Question	GLMM fixed effects	GLMM random effects (n categories)	GLMM fitting method	Estimates
Primary 1	Does mean coverage differ between arms at any of the six time-points?	arm + visit + year + arm × visit + arm × year + visit × year + arm × visit × year	District (9), ward (112), subvillage (659); visit-specific: district (54), ward (663); subvillage (2376); household (12999)	ML	Odds ratio estimate & Wald 95% CI at each survey visit in each year; LRT P-value for null hypothesis that each OR = 1.
Secondary 1	Does coverage differ between arms at any of the three Survey Visit 2 time-points?	Same as Primary 1	Same as Primary 1	ML	Odds ratio estimate & Wald 95% CI at survey visit 2 in each year (already calculated for Primary analysis 1); LRT P-value for null hypothesis that each OR = 1.
Secondary 2	What is the probability of coverage in a ward being below 40% in each arm, per trial month and averaged over the year.	arm + month + year + arm × month + arm × year	Same as Primary 1	ML	Distribution of predicted coverage by arm and month estimated by simulating new wards, incorporating model uncertainty via 10,000 parametric bootstrap samples; P(coverage < 40%) by arm and month & 95% CI
Secondary 3.1	Does the consistency of coverage over the duration of the trial differ between arms?	Same as Primary 1	Same as Primary 1	ML	Mean absolute difference between 6 consecutive time-points in log odds of predicted coverage exponentiated to an odds ratio, for each arm; the community:team ratio of odds ratios;

					95% CI for each ratio, estimated using 10,000 parametric bootstrap samples
Secondary 3.2	Does the rate of change in coverage from survey visit 1 to survey visit 2 differ between arms?	arm + visit + year + arm × visit + arm × year	Same as Primary 1	ML	arm × visit interaction odds ratio, Wald 95% CI and Wald P-value; Survey Visit effect odds ratio, Wald 95% CI and Wald P-value, for each arm.
Secondary 3.3	Given the observed rate of change of coverage within a year, what is the minimum coverage required at Survey Visit 1 to ensure that coverage at Survey Visit 2 does not drop below the critical vaccination threshold?	Same as Secondary 3.2	Same as Primary 1	ML	Coverage at survey visit 1 predicted to give 40% coverage at survey visit 2 & Wald 95% CI, calculated using the odds ratio estimates from Secondary Analysis 3.2.
Secondary 4	Does the consistency of coverage over space (i.e. between wards) differ between arms?	Same as Primary 1	Same as Primary 1, except that the inter-ward random effect variance varies between arms	MCMC	Median and 95% credible interval from the posterior distribution of the Community:Team ratio of inter-ward variances
