# Supplementary material for: A randomized controlled trial of the effectiveness of a community-based rabies vaccination strategy
Source: bioRxiv. 2024 Oct 31:2024.10.28.620430. Preprint. [Version 1] doi: 10.1101/2024.10.28.620430 (PMC11565783; doi:10.1101/2024.10.28.620430)
Supplement: Supplement 6 [file media-6.pdf]

Table S2. Estimates of fixed effects (log odds and log odds ratios) and random effects (variances) from the GLMMs fitted for the primary analysis. Numbers of observations, number of each random effect level, and marginal and conditional R^2 are also presented.

	Primary analysis null hypothesis model				Primary analysis alternative hypothesis model			
Predictors	Log-Odds	std. Error	CI	p	Log-Odds	std. Error	CI	p
(Intercept)	-0.60	0.32	-1.23, 0.02	0.060	-1.48	0.37	-2.19, -0.76	<0.001
Visit: V 2	-0.71	0.26	-1.22, -0.21	0.006	-1.11	0.32	-1.73, -0.49	<0.001
Year: Y 2	0.16	0.26	-0.35, 0.66	0.542	0.65	0.31	0.04, 1.25	0.036
Year: Y 3	0.90	0.26	0.40, 1.41	<0.001	1.11	0.31	0.51, 1.71	<0.001
VisitV2:YearY2	0.66	0.36	-0.05, 1.38	0.068	0.36	0.44	-0.50, 1.22	0.410
VisitV2:YearY3	-0.24	0.36	-0.95, 0.48	0.514	-0.14	0.44	-1.00, 0.72	0.751
Trial Arm: Community-based					1.73	0.31	1.11, 2.35	<0.001
VisitV2:Trial_ArmCommunity-based					0.79	0.35	0.10, 1.49	0.026
YearY2:Trial_ArmCommunity-based					-0.93	0.34	-1.61, -0.26	0.007
YearY3:Trial_ArmCommunity-based					-0.37	0.34	-1.04, 0.30	0.281
VisitV2:YearY2:Trial_ArmCommunity-based					0.56	0.49	-0.40, 1.52	0.256
VisitV2:YearY3:Trial_ArmCommunity-based					-0.21	0.49	-1.17, 0.75	0.669
**Random Effects**								
σ^2	3.29				3.29			
τ_{00}	3.68 Household.file.id				3.81 Household.file.id			
	1.04 sub_village.rnd				1.09 sub_village.rnd			
	2.30 sub_village				2.36 sub_village			
	0.47 ward.rnd				0.35 ward.rnd			
	1.25 ward				0.53 ward			
	0.15 district.rnd				0.16 district.rnd			
	0.46 district				0.58 district			
N	12999 Household.file.id				12999 Household.file.id			
	2376 sub_village.rnd				2376 sub_village.rnd			
	659 sub_village				659 sub_village			
	663 ward.rnd				663 ward.rnd			
	112 ward				112 ward			
	54 district.rnd				54 district.rnd			
	9 district				9 district			
Observations	18358				18358			
Marginal R^2 / Conditional R^2	0.017 / 0.744				0.079 / 0.751			
