# Supplementary material for: A randomized controlled trial of the effectiveness of a community-based rabies vaccination strategy
Source: bioRxiv. 2024 Oct 31:2024.10.28.620430. Preprint. [Version 1] doi: 10.1101/2024.10.28.620430 (PMC11565783; doi:10.1101/2024.10.28.620430)
Supplement: Supplement 7 [file media-7.pdf]

Table S3. Primary analysis (sensitivity analysis). Coverage and intervention odds ratio estimates (95% CI) at each survey estimated from the primary analysis GLMM re-fitted using a more stringent definition of coverage, where dogs claimed to be vaccinated but where no vaccination certificate could be produced were assumed to be unvaccinated (N dogs surveyed = 25677). The P-value is from a likelihood ratio test of the primary analysis null hypothesis of no intervention effect at any of the six surveys.

Year-visit	Coverage (95% CI)		Odds ratio (95% CI)	P-value
	Team	Community		
Y1-V1	0.18 (0.15, 0.21)	0.30 (0.27, 0.35)	2.00 (1.55, 2.58)	<0.001
Y1-V2	0.12 (0.10, 0.14)	0.24 (0.21, 0.28)	2.43 (1.89, 3.13)	<0.001
Y2-V1	0.22 (0.19, 0.26)	0.29 (0.25, 0.33)	1.39 (1.08, 1.80)	0.011
Y2-V2	0.18 (0.15, 0.21)	0.35 (0.31, 0.40)	2.53 (1.95, 3.29)	<0.001
Y3-V1	0.29 (0.25, 0.33)	0.39 (0.35, 0.43)	1.54 (1.19, 2.00)	0.001
Y3-V2	0.18 (0.15, 0.22)	0.31 (0.27, 0.35)	1.98 (1.53, 2.55)	<0.001
