# Supplementary material for: A randomized controlled trial of the effectiveness of a community-based rabies vaccination strategy
Source: bioRxiv. 2024 Oct 31:2024.10.28.620430. Preprint. [Version 1] doi: 10.1101/2024.10.28.620430 (PMC11565783; doi:10.1101/2024.10.28.620430)
Supplement: Supplement 8 [file media-8.pdf]

Table S4. Estimates of fixed effects (log odds and log odds ratios) and random effects (variances) from the GLMMs fitted for the primary analysis. The primary analysis GLMMs were re-fitted using a more stringent definition of coverage, where dogs claimed to be vaccinated but where no vaccination certificate could be produced were assumed to be unvaccinated. Numbers of observations, number of each random effect level, and marginal and conditional R^2 are also presented.

	Primary analysis null hypothesis model				Primary analysis alternative hypothesis model			
Predictors	Log-Odds	std. Error	CI	p	Log-Odds	std. Error	CI	p
(Intercept)	-2.39	0.24	-2.86, -1.91	<0.001	-3.96	0.29	-4.53, -3.38	<0.001
Visit: V 2	-0.85	0.30	-1.43, -0.27	0.004	-1.30	0.31	-1.91, -0.69	<0.001
Year: Y 2	0.16	0.29	-0.41, 0.73	0.580	0.70	0.32	0.08, 1.33	0.028
Year: Y 3	0.95	0.29	0.37, 1.52	0.001	1.65	0.32	1.01, 2.28	<0.001
VisitV2:YearY2	0.89	0.41	0.07, 1.70	0.033	0.57	0.44	-0.30, 1.44	0.199
VisitV2:YearY3	-0.15	0.41	-0.96, 0.66	0.710	-0.30	0.44	-1.17, 0.58	0.506
Trial Arm: Community-based					1.81	0.34	1.14, 2.47	<0.001
VisitV2:Trial_ArmCommunity-based					0.51	0.44	-0.35, 1.37	0.245
YearY2:Trial_ArmCommunity-based					-0.94	0.44	-1.81, -0.07	0.034
YearY3:Trial_ArmCommunity-based					-0.67	0.44	-1.54, 0.19	0.127
VisitV2:YearY2:Trial_ArmCommunity-based					1.04	0.63	-0.19, 2.27	0.096
VisitV2:YearY3:Trial_ArmCommunity-based					0.13	0.62	-1.09, 1.35	0.833
**Random Effects**								
σ^2	3.29				3.29			
τ_{00}	7.90 $_{\text{Household.file.id}}$				11.95 $_{\text{Household.file.id}}$			
	1.14 $_{\text{sub\_village.rnd}}$				1.72 $_{\text{sub\_village.rnd}}$			
	1.77 $_{\text{sub\_village}}$				2.27 $_{\text{sub\_village}}$			
	0.59 $_{\text{ward.rnd}}$				0.80 $_{\text{ward.rnd}}$			
	0.65 $_{\text{ward}}$				0.00 $_{\text{ward}}$			
	0.22 $_{\text{district.rnd}}$				0.00 $_{\text{district.rnd}}$			
	0.00 $_{\text{district}}$				0.00 $_{\text{district}}$			
N	18067 $_{\text{Household.file.id}}$				18067 $_{\text{Household.file.id}}$			
	2473 $_{\text{sub\_village.rnd}}$				2473 $_{\text{sub\_village.rnd}}$			
	687 $_{\text{sub\_village}}$				687 $_{\text{sub\_village}}$			
	663 $_{\text{ward.rnd}}$				663 $_{\text{ward.rnd}}$			
	112 $_{\text{ward}}$				112 $_{\text{ward}}$			
	54 $_{\text{district.rnd}}$				54 $_{\text{district.rnd}}$			
	9 $_{\text{district}}$				9 $_{\text{district}}$			
Observations	25677				25677			
Marginal R^2 / Conditional R^2	0.017 / 0.792				0.060 / 0.846			
